# Evaluation of HDL-modulating interventions for cardiovascular risk reduction using a systems pharmacology approach[S]

**DOI:** 10.1194/jlr.M057943

**Published:** 2016-01

**Authors:** Kapil Gadkar, James Lu, Srikumar Sahasranaman, John Davis, Norman A. Mazer, Saroja Ramanujan

**Affiliations:** Genentech Research and Early Development,* South San Francisco, CA; Roche Pharma Research and Early Development,† Clinical Pharmacology, Disease Modeling Group, Roche Innovation Center Basel, Basel, Switzerland

**Keywords:** high density lipoprotein, apolipoprotein A-I, low density lipoprotein, cholesterol metabolism, cholesteryl ester transport protein, reverse cholesterol transport, in-silico model

## Abstract

The recent failures of cholesteryl ester transport protein inhibitor drugs to decrease CVD risk, despite raising HDL cholesterol (HDL-C) levels, suggest that pharmacologic increases in HDL-C may not always reflect elevations in reverse cholesterol transport (RCT), the process by which HDL is believed to exert its beneficial effects. HDL-modulating therapies can affect HDL properties beyond total HDL-C, including particle numbers, size, and composition, and may contribute differently to RCT and CVD risk. The lack of validated easily measurable pharmacodynamic markers to link drug effects to RCT, and ultimately to CVD risk, complicates target and compound selection and evaluation. In this work, we use a systems pharmacology model to contextualize the roles of different HDL targets in cholesterol metabolism and provide quantitative links between HDL-related measurements and the associated changes in RCT rate to support target and compound evaluation in drug development. By quantifying the amount of cholesterol removed from the periphery over the short-term, our simulations show the potential for infused HDL to treat acute CVD. For the primary prevention of CVD, our analysis suggests that the induction of ApoA-I synthesis may be a more viable approach, due to the long-term increase in RCT rate.

Large epidemiological studies in multiple populations have consistently shown that high levels of LDL cholesterol (LDL-C), as well as low levels of HDL cholesterol (HDL-C), are associated with increased CVD risk (1). These relationships have been attributed, in part, to the role of LDL in depositing cholesterol in atherosclerotic plaques and promoting inflammation, and the role of HDL in reverse cholesterol transport (RCT), the process by which cholesterol is taken up by HDL particles and transported to the liver for subsequent excretion. A causative role of LDL in CVD risk has been supported by the success of LDL-lowering therapy with statins, currently the most prescribed class of drugs for the treatment of hypercholesterolemia and atherosclerosis. Due to the success of statins, additional LDL-lowering therapeutic strategies, such as PCSK9 antagonism, are being actively pursued in pharmaceutical development (2). However, to date, no HDL-targeted therapies have been shown to decrease CVD risk (1). Notably, the development of two inhibitors of cholesteryl ester transport protein (CETP) were halted due to adverse events and a lack of efficacy in large phase III trials, despite large increases in HDL-C levels (3, 4). Proposed hypotheses for the lack of efficacy include an imperfect role of total HDL-C as a biomarker of RCT-mediated plaque reduction, possibly due to a differential impact of larger versus smaller HDL particles on CVD risk and/or the existence of separate classes of beneficial and inflammatory HDL (1). Despite the recent failures of compounds with specific mechanisms of action, HDL-modifying strategies continue to be of interest.

A number of potential HDL-elevating therapies are currently under active investigation. A direct approach is the infusion of reconstituted HDL (rHDL), which has been shown to increase HDL with beneficial effects on plaque burden (5, 6); one rHDL formulation is currently under clinical development for the treatment of acute coronary syndrome (7). Delipidation is another approach to HDL modulation, whereby circulating HDL is extracted, depleted of cholesteryl ester (CE), and subsequently reintroduced into the blood in serial autologous infusions in a short in-patient procedure (8). Another target for increasing HDL is the transporter, ABCA1. ABCA1 is thought to initiate the cholesterol loading of lipid-poor ApoA-I, leading to subsequent maturation of HDL particles (9, 10). The critical role of ABCA1 in HDL formation is evidenced by low HDL-C levels in heterozygous and homozygous patients of Tangier disease with loss-of-function mutations in the ABCA1 gene (11). In nonhuman primates (NHPs), suppression of microRNAs, miR-33a and miR-33b, have been shown to increase hepatic expression of ABCA1, leading to increases in the HDL level (12). Finally, another recently investigated approach for raising HDL is to increase the plasma level of ApoA-I, the main protein constituent and precursor of HDL. Due to the central role of lipid-poor ApoA-I in initiating RCT via ABCA1-mediated lipidation, it is thought that increasing synthesis of ApoA-I will promote generation of additional HDL and hence increase RCT (13). The small molecule, BET bromodomain antagonist RVX-208, which has been shown to increase the production of ApoA-I in monkeys and humans, is currently under clinical development. Given the continued interest in HDL modulation, a greater understanding of how these different therapeutic approaches might influence RCT, plaque burden, and ultimately CVD risk is needed (14).

Quantitative systems modeling is an approach that is being increasingly applied to pharmaceutical drug development, from target identification and validation, to compound design and translational and clinical trial planning (15). In contrast to traditional pharmacokinetic pharmacodynamic modeling, systems models frequently integrate heterogeneous data from diverse sources into a mechanistic mathematical model of the underlying biological system of interest (16). The approach incorporates multi-scale phenomena, ranging from molecular events to physiological feedbacks, to allow in silico investigation of the effects of therapies on pathophysiological processes. In the context of HDL metabolism, where there is inadequate understanding of the relationship between HDL-C and CVD risk, systems modeling provides a unique approach to investigate the potential impact of HDL-modifying strategies. By linking target pathways to multiple HDL-related measures including HDL-C, particle concentration, and particle size, and subsequently to the difficult-to-assess RCT rate, systems modeling provides a unifying framework for evaluating and comparing multiple therapeutic approaches. In this study, we apply a quantitative systems modeling approach to predict the relative impact of these various HDL-modifying approaches on ApoA-I, HDL-C, and RCT, and qualitatively compare predictions to available data on modification of atherosclerotic plaque load.

Lu et al. (17) developed an in silico model of lipoprotein metabolism and kinetics (LMK) that relates the dynamics of ApoA-I, CE loading onto HDL, and HDL remodeling to common experimental measures, including mean ApoA-I, HDL-C, and HDL particle concentration and size, and ultimately to RCT rate. In the LMK model, the RCT rate is quantified as the total ABCA1-mediated efflux of cholesterol into plasma HDL originating from both peripheral tissues and the liver, and is mathematically represented as a function of available lipid-poor ApoA-I particles, the number of cholesterol molecules loaded onto each nascent particle, and ABCA1 activity. The model was calibrated, in part, to clinical data from subjects with CETP mutations and validated against clinical data from subjects with ApoA-I and ABCA1 mutations. It was then used to simulate the effects of CETP inhibition and ABCA1 upregulation, predicting results consistent with clinical observations, including a negligible impact of CETP inhibition on RCT flux despite a significant increase in HDL-C, and a positive impact on RCT flux for ABCA1 upregulation. The systems model developed in this work enables predictions of entities such as lipid-poor ApoA-I and RCT rate that were not directly measured either in the calibration or validation studies; hence, the predictions made by the model across the different interventions examined provide value in addition to the measurements that were available from the clinical studies alone. The ability of the LMK model to recapitulate the clinical data on impact of mutations, to make clinically consistent predictions for CETP inhibition, and to reproduce the biphasic behavior seen in results of tracer kinetic studies on ApoA-I together provide confidence in its suitability for investigating the kinetics of the HDL-related interventions studied here (17). While the model only includes mean HDL particle measures (e.g., ApoA-I concentration, HDL-C, HDL size, and particle concentration), unlike the heterogeneous particle model of (18), it correctly accounts for the relationship between particle size and the number of ApoA-I molecules per particle (19). Lastly, in contrast to the model of van de Pas et al. (20), which quantifies the amount of cholesterol in various organs and compartments, the LMK model enables the quantification of the whole-body in vivo RCT rate.

In this work, we use the LMK model as a platform to study a number of additional therapeutic interventions. The results provide novel simulation-based predictions on the impact of upregulation of ApoA-I synthesis by RVX-208 (13), administration of CSL-111 rHDL (6), and infusion of delipidated HDL (21). We predict and compare the quantitative impact of these interventions on ApoA-I, HDL-C, HDL size, and RCT, and contrast these predictions with the simulated effects of ABCA1 upregulation previously studied in (17). The predicted changes in RCT rate are also qualitatively compared with the available data on the effects of these interventions on plaque burden to confirm the concordance of the predicted RCT changes with the observed effect on an established biomarker of CVD.

## METHODS

In the current work, we evaluate the following HDL modifying therapies that target the RCT pathway: *1*) upregulation of ApoA-I synthesis; *2*) infusion of rHDL; *3*) infusion of delipidated HDL; and *4*) upregulation of ABCA1 [previously studied in Lu et al. (17)]. The virtual population used in the current analysis is identical to the 1,000 virtual subjects utilized to predict the mean response and 95% confidence interval (CI) for the interventions of CETP inhibition and ABCA1 upregulation in the LMK model (17). In particular, the virtual subjects were sampled from a multivariate normal distribution whose covariance matrix corresponds to the Bayesian posterior. From the generated set of virtual subjects, the criteria Δχ^2^ ≤ 42.557 (corresponding to 29 degrees of freedom) was applied to select those that fell within the 95% CI.

### Upregulation of ApoA-I synthesis

**Fig. 1.**
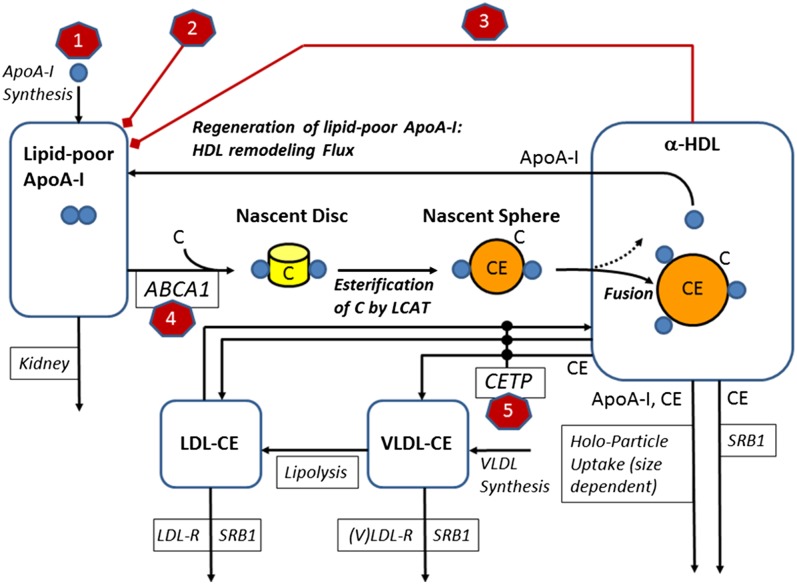
A schematic diagram of the LMK model with a depiction of the set of HDL interventions that have been analyzed: *1*) upregulation of ApoA-I synthesis; *2*) infusion of rHDL; *3*) infusion of delipidated HDL; *4*) upregulation of ABCA1; and *5*) inhibition of CETP.

In the absence of pharmacokinetic data for RVX-208, the drug effect is represented as a constant relative upregulation of ApoA-I synthesis (see marker 1 in **Fig. 1**). The extent of upregulation by RVX-208 was calibrated to data reported from an efficacy study with RVX-208 in statin-treated patients with stable coronary artery disease (22). The study included three different active dose groups: 50 mg, 100 mg, and 150 mg given twice daily for 12 weeks. The median percent increase in HDL-C reported for the three groups (3.2, 6.3, and 8.3%, respectively) was used to calibrate the effect of the therapy on the degree of upregulation for ApoA-I synthesis. The model was then used to predict the accompanying changes in ApoA-I, LDL-C, HDL particle size, and RCT flux.

### rHDL infusions

For these investigations, we simulated treatment with CSL-111, a rHDL material composed of ApoA-I and phosphatidylcholine in a molar ratio of 1:150. Using molecular masses of 786 and 28,500 Da for ApoA-I and phospholipid, respectively, a dose of 80 mg/kg of CSL-111 corresponds to an equivalent infusion of 34.6 mg/dl of lipid-free ApoA-I into the plasma. The intervention was modeled by adding the ApoA-I content of the infusion into the lipid-poor ApoA-I pool in the LMK model; no additional model adjustments or calibration were performed.

### Infusion of delipidated HDL

Serial autologous infusions of selective HDL-delipidated plasma have been explored clinically (21). In a clinical study, 14 patients underwent the selective HDL delipidation process of 1 l of plasma every 7 days, for a total of seven treatment procedures. In the model, this protocol is represented by the removal of a fraction of α-HDL particles, followed by the introduction of the corresponding ApoA-I content into the lipid-poor pool (refer to marker 3 in Fig. 1). With the selective delipidation process, lipid-poor ApoA-I is shown to increase from 5.6 to 79.1%, whereas HDL-C is predicted to decrease from 92.8 to 20.9%. This selective delipidation procedure was simulated in the virtual patients to evaluate the impact of the process on RCT rate. The representation of this HDL modifying therapy included implementing direct effects on lipid-poor ApoA-I and HDL so as to match the reported magnitudes; no additional model adjustments or calibration were performed.

### ABCA1 upregulation

For comparison with the other interventions, we simulated ABCA1 upregulation as per the original publication (17). This was performed by increasing the rate constant *k*_ABCA1_ (marker 4 of Fig. 1; refer to the model equations of the LMK model in the online supplement). Although ABCA1 protein is not explicitly represented in the model, increasing the rate constant increases the RCT flux that would result from an increased abundance of ABCA1 protein. The LMK model has assumed a linear dependence on the levels (or activity) of ABCA1, which is supported by ABCA1 mutation data, but is an extrapolation for ABCA1 upregulation.

## RESULTS

### Upregulation of ApoA-I synthesis

**Fig. 2.**
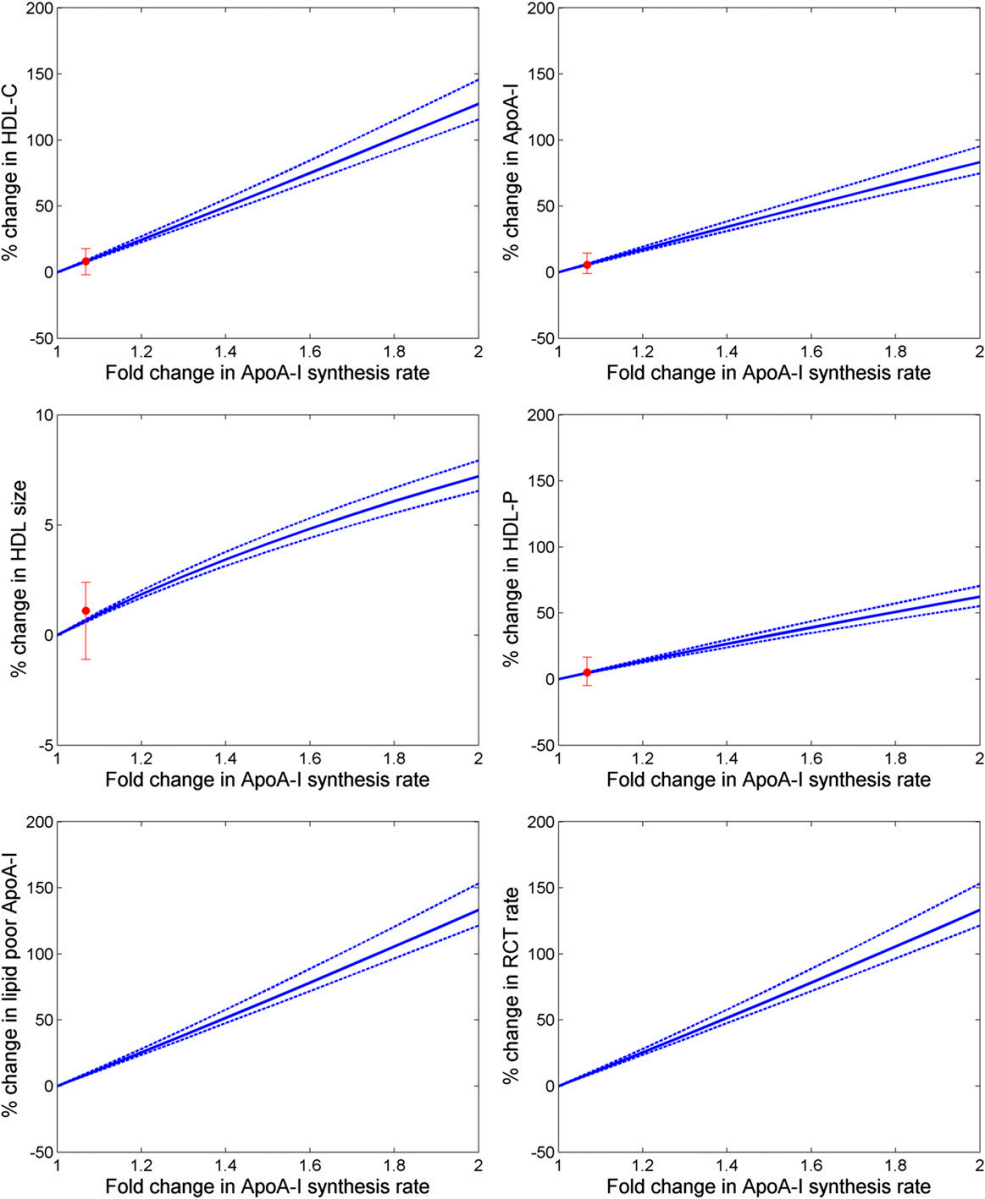
Changes in lipid parameters and RCT under the upregulation of ApoA-I synthesis; solid line is the mean of population and the dotted lines are the 5–95% CI of the mean; the solid red circle with error bars shows the median and interquartile range of the clinical data for RVX-208 at the 150 mg dose reported in (22).

To understand and predict the impact of upregulation of ApoA-I synthesis, we simulated a 2-fold increase in the ApoA-I synthesis rate. Results shown in **Fig. 2** illustrate a greater than 100% increase in HDL-C, as well as an increase in ApoA-I of up to 75%. More importantly, the model predicts an increase of RCT rate in excess of 100%. The result that the fold increases for both HDL-C and RCT rate are higher than that for ApoA-I synthesis rate is due to the fact that the model describes ApoA-I recycling as a consequence of the nonlinear relationship between HDL size and the number of ApoA-I molecules on HDL particles. We then simulated the impact of the ApoA-I-upregulating compound, RVX-208, by specifying a 6.8% upregulation of ApoA-I synthesis (shown in **Table 1**) that was calibrated to match the percentage of HDL-C increases at the highest dose of 150 mg reported in (22). A comparison of conventional lipid measures reported with those obtained from the simulation, also shown in Table 1, confirms good agreement between the reported and model-predicted results for ApoA-I, particle concentration, and particle size by 150 mg RVX-208. The corresponding increase in RCT rate was predicted to be 8.5%.

**TABLE 1.** Comparison of the lipid measures reported in (22) and model simulations of the LMK model

	RVX-208 (150 mg Dose)	
	Reported Data	Model Simulation
Upregulation of ApoA-I synthesis (%)	NA	6.8
Change in HDL-C (%)	8.3 (−1.9 to 17.9)	8.3 (7.6–9.1)
Change in ApoA-I (%)	5.6 (−1.1 to 14.3)	5.9 (5.4–6.6)
Change in HDL particle concentration (%)	5.1 (−4.8 to 16.5)	4.8 (4.3–5.2)
Change in HDL particle size (%)	1.1 (−1.1 to 2.4)	0.7 (0.6–0.7)
Change in LDL-C (%)	1.0 (−14.2 to 10.1)	8.4 (7.8–9.3)
Change in RCT rate (%)		8.5 (7.9–9.4)

Clinical data are given with their median and interquartile range; simulation results are given with mean and 95% CI of mean .

### rHDL infusions

**Fig. 3.**
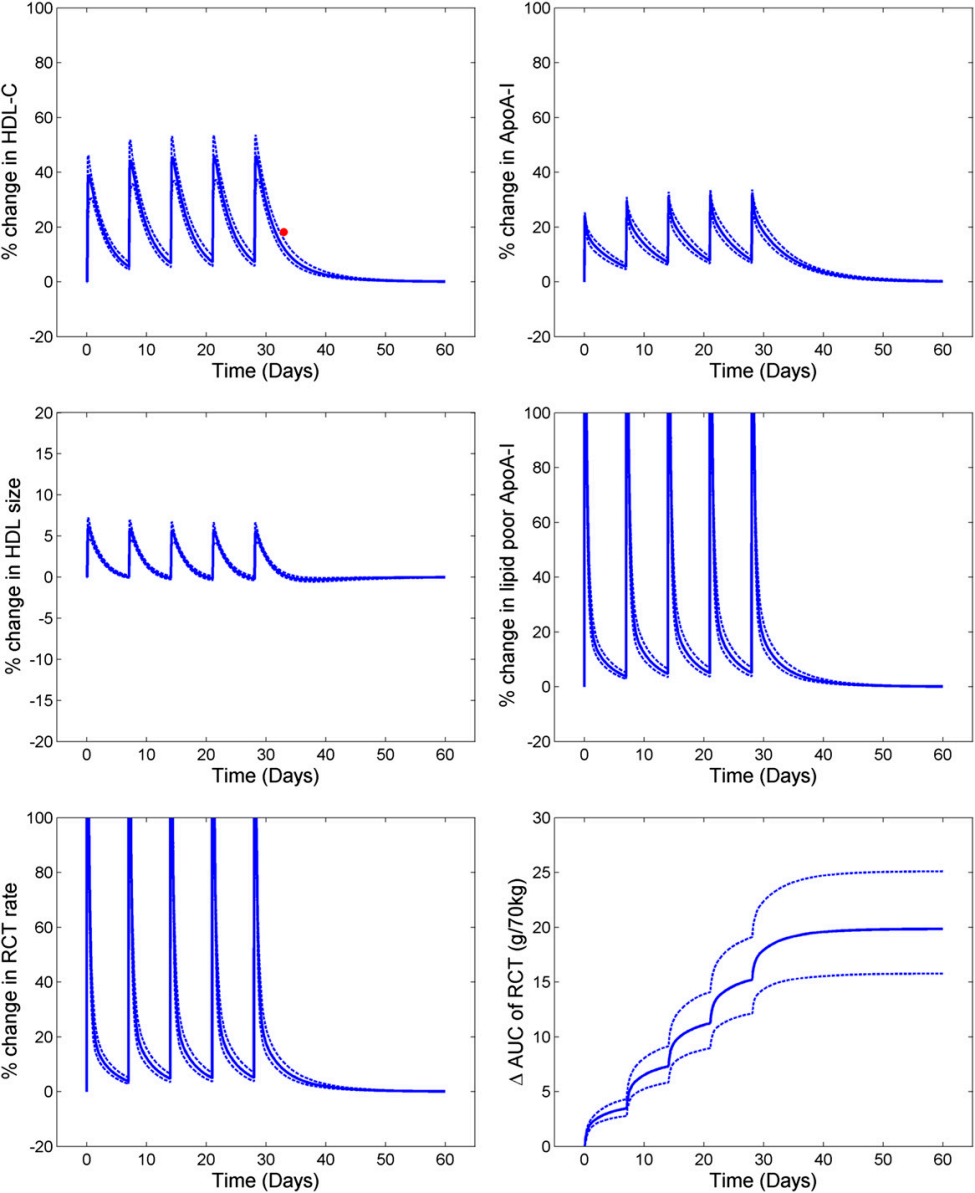
Simulated profiles of lipid parameters and RCT for CSL-111; solid line is the mean of population and the dotted lines are the 5–95% CI of the mean. The solid red circle shows clinical data (6) where available.

**Figure 3** shows the simulated profiles for the lipid measures and RCT for the five once-weekly infusions of CSL-111 at a dose of 80 mg/kg (**Table 2**). With each infusion, there is an immediate increase in lipid-poor ApoA-I concentration that results in an increase in RCT flux. There is also a subsequent increase in HDL-C and HDL particle size. The excess cholesterol taken up into plasma, as indicated by the area under the curve of RCT, is shown in Fig. 3. The reported HDL-C increase 5 days after the last infusion was 15–20%, in good agreement with the 12.3% increase predicted by the model. The simulation results indicate that the total amount of cholesterol entering the plasma compartment after five doses of rHDL exceeds the mass of ApoA-I infused. While this may appear counterintuitive, because the mass of ApoA-I in human plasma is several times that of HDL-C, the prediction reflects the fact that each ApoA-I molecule can be utilized several times in cholesterol transport as a result of the cycling of the infused ApoA-I between the α-HDL and lipid-poor ApoA-I pools. Consistent with this predicted result, clinical administration of 4 g of pro-ApoA-I in liposomal form reportedly resulted in an approximately 5 g increase in RCT, as measured by fecal steroid excretion (23). In the case of CSL-111, neglecting intermediate remodeling steps between the infused rHDL and endogenous HDL and using a simplified representation of the injected ApoA-I dose as lipid-poor ApoA-I appears to be sufficient. In the LMK model, the cholesterol-holding capacity for CSL-111 particles is assumed to be the same as that of the endogenously synthesized lipid-poor ApoA-I particles. The phospholipid layer is not explicitly represented in the model for the lipid-poor ApoA-I and α-HDL particles, and it is assumed that the cholesterol-holding capacity is not altered based on phospholipid content. CSL-111 has recently been superseded by a different rHDL formulation, CSL-112, for which single and multiple ascending dose data have recently been published (7). However, because of the reported extensive in vivo remodeling of CSL-112, representation of this therapy is not included in the current work; as more quantitative data on these processes become available, they may be used to support the implementation of this therapy.

**TABLE 2.** Comparison of the changes in lipid measures reported in (6) and model simulations using the LMK model for CSL-111 infusions

	CSL-111 (80 mg/kg Dose)	
	Reported Data	Model Simulation
Change in LDL-C (%)	14.3	13.5 (11.2–16.8)
Change in HDL-C (%)	18.2	12.3 (10.2–15.5)

Clinical data are given as percent change in mean values (pre-infusion and 5–7 days post-infusion) and the model predictions are given with mean and 95% CI of the mean.

### Infusion of delipidated HDL

**Fig. 4.**
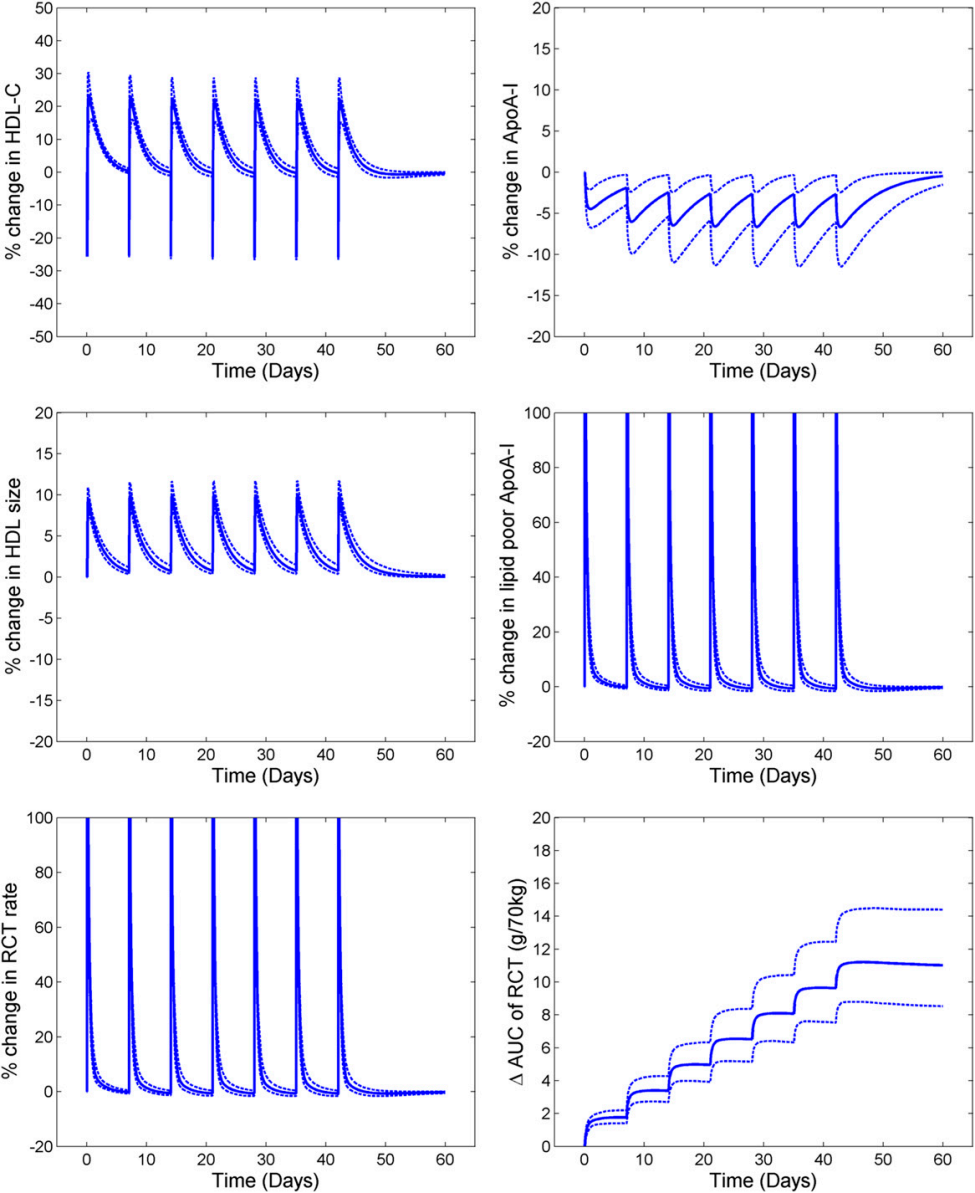
Simulated profiles of lipid parameters and RCT for selective HDL delipidation; solid line is the mean of population and the dotted lines are the 5–95% CI of the mean.

**Table 3** shows the comparison of clinical data and the model simulations for the pre- and postprocedure lipid profiles of 1 l of plasma undergoing the delipidation process. **Figure 4** shows the simulated profiles for the outputs of HDL, lipid-poor ApoA-I, total ApoA-I, and RCT rate for seven consecutive delipidation procedures performed weekly. With each delipidation there is a ∼25% drop in HDL-C. The ApoA-I (total) is unaltered, but appears in the lipid-poor ApoA-I form instead of ApoA-I on α-HDL. This explains the large percentage increase in lipid-poor ApoA-I, but no change in ApoA-I (total). Following the delipidation process, there is small drop in ApoA-I due to the faster clearance of the lipid-poor particles compared with the α-HDL particles. Because lipid-poor ApoA-I has an increased capacity to take up cholesterol from the periphery, this results in an immediate increase in the RCT rate. The lipid-poor ApoA-I particles that take up the cholesterol then mature into α-HDL particles, resulting in a subsequent increase in HDL-C shortly following the procedure. The increased RCT fluxes are predicted to be short-lived, however, with the time-averaged increase in RCT rate during the whole treatment procedure predicted to be 7.3%. Furthermore, simulation results predict that ApoA-I levels drop during the treatment period due to the shift of ApoA-I from the α-HDL to the lipid-poor ApoA-I pool and to the fact that the clearance of lipid-poor ApoA-I by the kidney is substantially greater than the clearance of α-HDL particles by the holo-particle uptake mechanism.

**TABLE 3.** Comparison of the lipid measures reported in (21) with model simulations using the LMK model corresponding to a delipidation procedure of 1 l of blood

	Reported Data		Model Simulation	
	Prior to Delipidation	After Delipidation	Prior to Delipidation	After Delipidation
Lipid-poor ApoA-I (%)	5.6	79.1	4.5 (2.8–7.6)	80.9 (80.6–81.5)
α-HDL (%)	92.8	20.9	95.5 (92.4–97.2)	19.1 (18.5–19.4)

### ABCA1 upregulation

**Fig. 5.**
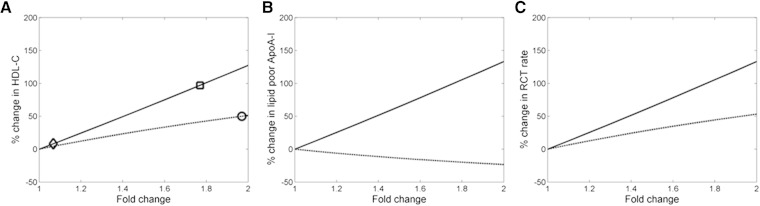
Mean response of virtual population for ApoA-I upregulation (solid line) and ABCA1 upregulation (dashed line). A: HDL-C increase with anti-miR-33 with NHP (circle), HDL-C increase with RVX-208 in NHP (square), and HDL-C with highest tested dose of 150 mg BID in humans with RVX-208 (diamond). B: Lipid-poor ApoA-I. C: RCT rate.

**Figure 5** shows the mean responses of the virtual population to the upregulation of either ApoA-I synthesis or ABCA1 expression. For a given fold increase in the target, the predicted increase in RCT rate for the upregulation of ABCA1 is lower than that for ApoA-I synthesis. This interesting and perhaps nonintuitive finding reflects the fact that the impact of ABCA1 upregulation is limited by the availability of the substrate, lipid-poor ApoA-I. ABCA1 upregulation depletes lipid-poor ApoA-I by driving its conversion to α-HDL particles; whereas, increasing the synthesis of new ApoA-I protein results in a parallel increase in lipid-poor ApoA-I. In NHPs, the subcutaneous delivery of anti-miR-33 has been shown to increase the expression of ABCA1 and to induce an up to 50% increase in plasma HDL-C (12). Based on the simulation analysis performed for the virtual population, this 50% increase in HDL-C corresponds to an approximately 2-fold increase in ABCA1 expression. In comparison, a NHP study of RVX-208 increased HDL-C by 97% at the highest tested dose of 60 mg/kg/day (24), for which the model predicts a roughly 1.75-fold increase in ApoA-I synthesis. Hence, under the assumption of linear kinetics, our results show the relative sensitivity of HDL-C to comparable levels of upregulation of ApoA-I synthesis versus ABCA1.

## DISCUSSION AND CONCLUSIONS

The inverse relationship between CVD risk and HDL-C levels observed in epidemiological studies led to the hypothesis that HDL-C-raising therapies would be atheroprotective. However, the recent failures of some HDL-C-raising compounds to impact CVD risk have raised the concern that HDL-C levels bear a correlational rather than a causal relationship to CVD risk. In light of this recent data, the focus of HDL-targeted therapeutic approaches has shifted away from raising HDL-C to the upregulation of the RCT pathway. With the current attention on RCT as an important aspect of HDL function, the ability to quantify or predict changes in RCT rate with therapy takes on an increased importance.

In this work, we have applied a systems pharmacology approach utilizing the previously described LMK model (17) to evaluate several HDL-targeted interventions by mechanistically linking their impact on HDL properties to the corresponding changes in the RCT rate. All the interventions studied herein (upregulation of ApoA-I synthesis or ABCA1 expression; infusion of either delipidated or rHDL) were predicted to increase both HDL-C and the RCT rate, representing a qualitatively different result from the increased HDL-C and slightly decreased RCT rate previously predicted for CETP inhibition (17). The different predicted effects on RCT rates between CETP inhibition and the interventions studied here can be explained by differences in their mechanisms of action. CETP inhibition increases HDL-C by blocking the clearance of HDL-CE, while the other approaches work on increasing the flux of cholesterol into HDL. While CETP inhibition can increase HDL-C to a greater extent than the other interventions, it also greatly increases mean particle size. As a result of blocking the CETP-mediated pathway for CE elimination from HDL particles, there is a negative impact on the rate of HDL core reduction, a process that contributes to the regeneration of lipid-poor ApoA-I. Hence, CETP inhibition is predicted to result in CE-rich HDL particles and potentially a decrease in lipid-poor ApoA-I, both of which could lead to a reduction in cholesterol uptake capacity. Here it is important to note that the LMK model predicts that CETP inhibition does not increase the RCT rate on a whole-body level. However, it has been shown that strong CETP inhibition lowers LDL-C appreciably (also consistent with the predictions of the LMK model). Therefore, we cannot rule out the possibility that strong CETP inhibition might reduce CVD risk for this reason. In contrast, the interventions studied here increase the rate of cholesterol loading onto lipid-poor ApoA-I mediated by ABCA1. These results support the hypothesis that HDL-C levels alone are insufficient to fully characterize the RCT rate, and by extension, RCT-mediated effects on plaque accumulation and CVD risk. Rather, measurements of HDL particle size distribution, particle concentration, and total HDL-C are all important for understanding the likely net impact of HDL-modifying treatment on RCT.

The model results illustrate that despite the failures to date of CETP inhibitors and the apparent inadequacy of HDL-C as a marker of RCT rate, modulation of HDL-related targets may still be a viable approach to reducing plaque load and CVD risk. Our model predicts that infusions of rHDL or HDL delipidation can give rise to large increases in HDL-C and transient increases in RCT rates. These interventions may be more appropriate as intensive acute treatments. For longer-term treatments of chronic CVD, upregulation of ApoA-I synthesis is predicted to give a sustained increase in RCT rate and may be more effective in terms of the total cholesterol removed over longer time-frames.

In the description of the lipidation of lipid-poor ApoA-I by the ABCA1 transporter, the LMK model assumes nonsaturable linear kinetics with respect to the concentration of ApoA-I. Under this assumption, we can adequately predict the HDL-C increases under CSL-111 treatment (see Fig. 3). Furthermore, the linearity of the rate law is consistent with the ApoA-I and ABCA1 mutation data. Supposing that the linear kinetics assumption can be extrapolated, we predict that RCT rate could be less sensitive to ABCA1 upregulation as a therapeutic strategy than to the induction of ApoA-I synthesis, because ABCA1 upregulation is predicted to lead to the depletion of lipid-poor ApoA-I, mitigating some of the positive impact on RCT. As a result of this depletion, RCT rate is predicted to increase less than proportionally to the degree of ABCA1 upregulation, highlighting a potentially important consideration with respect to the development of ABCA1 targeted therapies. This model prediction highlights a difference between ABCA1- and ApoA-I-based approaches and suggests the need for further experimentation to verify or refute it. For the interventions evaluated with the LMK model in this work, the model predictions have been compared with the clinical data wherever available (Tables 1, 2; Figs. 2, 3) for the different interventions evaluated in the model. Further validation of the model predictions would be possible as and when clinical data for the interventions tested become publicly available.

It should be recognized that the simplifying mechanistic assumptions used in the LMK model result in some limitations on the accurate representation of some of the interventions investigated in this work. As an example, the suboptimal esterification of the cholesterol that is mobilized by the therapeutic intervention, CER-001 (25), cannot be assessed with the LMK model due to the assumption of complete and rapid esterification of cholesterol to CE. Furthermore, if the remodeling of infused rHDL particles is different from the endogenously generated HDL particles, they would have to be studied and modeled. This limitation precludes the use of the current model to predict infusions of CER-001 due to the presence of negatively charged phospholipid, which has been shown to prevent the fusion of CER-001 with endogenous HDL particles. Similarly, for CSL-112, the remodeling of the rHDL particles has not been included in the current model and, thus, any simulations for this intervention are out of the scope of the model. In the interpretation of the simulation results, one should be aware of a number of modeling assumptions made regarding cholesterol metabolism. In particular, in the simulation of large doses of rHDL infusions, there could potentially be a saturation of CETP in the transfer of CE between HDL and LDL/VLDL, while the model assumes linear rates. Furthermore, additional details of LDL metabolism are needed to accurately predict the corresponding levels of LDL-C and ApoB, as recently shown (26). In order to address these and other questions, further extensions of the LMK model would be needed.

It is also important to bear in mind that while we have used the LMK model to elucidate the links between HDL targeted therapies and changes in RCT rate, the quantitative link between RCT and CVD risk remains to be established. Because clinical trials evaluating the impacts of therapies on CVD can be long and expensive, surrogate markers that can reliably predict cardiovascular outcomes would be highly valuable. A potential surrogate marker of clinical efficacy is the coronary artery plaque volume derived from intra-vascular ultrasound (IVUS). The available IVUS data on plaque load modification by therapies considered in this work, presented in **Table 4**, shows that for interventions other than CETP inhibitors, there is a trend for reducing plaque volume (relative to control). These observations are consistent with our predictions on RCT impact: CETP inhibition was predicted to result in an insignificant change in RCT, while all other interventions studied were predicted to increase RCT. However, a quantitative link between RCT and plaque burden is currently out of scope for the LMK model. Also, additional work would be needed to understand the variability in the patient response in the various imaging studies. Some modeling efforts [e.g., (14)] have attempted to link changes in plasma lipids to macrophage recruitment and foam cell formation, and ultimately to plaque volume and geometry; further effort will be needed to incorporate RCT rate into such plaque models, as well as to relate plaque predictions to cardiovascular outcomes.

**TABLE 4.** Published IVUS data for the interventions explored in this work and the predictions for RCT

Intervention	Study Arm	Change in PAV	Change in AV (mm^3^)	Change in AV (%)	Reference
RVX-208	RVX-208 at 100 mg bid for 26 weeks (n = 243)	−0.4 (median)	−4.2	—	ASSURE study (22)
	Placebo for 26 weeks (n = 80)	−0.3 (median)	−3.8	—	
CSL-111	CSL-111 at 4 weekly infusions of 40 mg/kg (n = 89)	—	−5.34 (median)	−3.41 (median)	ERASE study (27)
	Placebo (n = 47)	—	−2.33 (median)	−1.62 (median)	
Delipidation	HDL delipidation, seven plasmapheresis 1 week apart (n = 14)	—	−12.18 (mean)	—	(21)
	Control (n = 12)	—	2.8 (mean)	—	
CETP inhibitor	Torcetrapib + atorvastatin	0.12 (mean)	—	—	ILLUSTRATE (28)
	Atorvastatin	0.19 (mean)	—	—	

AV, atheroma volume; PAV, percent atheroma volume.

In summary, we have shown that the use of a systems pharmacology model provides a valuable way to integrate prior knowledge to predict potential impact of HDL-modulating therapies, especially given the challenges of measuring RCT, the critical process behind the proposed beneficial impact of HDL. Because the systems model links the different targets to measurable HDL-related biomarkers and ultimately to predicted RCT, it also provides guidance on which measurements will inform further evaluation of each target or compound. As more clinical data are collected, parameters and assumptions of the systems model can be refined and help to support drug development applications, such as evaluating new treatment approaches, understanding patient variability, and proposing optimal pairing of patients and treatment modality.

## Supplementary Material

Supplemental Data
