# Supplementary material for: Evaluation of HDL-modulating interventions for cardiovascular risk reduction using a systems pharmacology approach
Source: J Lipid Res. 2016 Jan;57(1):46–55. doi: 10.1194/jlr.M057943 (PMC4689335; doi:10.1194/jlr.M057943)
Supplement: Supplemental Data [file supp_57_1_46__index.html]

Evaluation of HDL modulating interventions for cardiovascular risk reduction using a systems pharmacology approach — Evaluation of HDL-modulating interventions for cardiovascular risk reduction using a systems pharmacology approach — Supplemental Data 

# Evaluation of HDL-modulating interventions for cardiovascular risk reduction using a systems pharmacology approach

## Supplemental Data

- supplement (.pdf, 805 KB) - supplement
