# Supplementary material for: Evaluation of HDL-modulating interventions for cardiovascular risk reduction using a systems pharmacology approach
Source: J Lipid Res. 2016 Jan;57(1):46–55. doi: 10.1194/jlr.M057943 (PMC4689335; doi:10.1194/jlr.M057943)
Supplement: Supplemental Data [file 10.1194_M057943_jlr.M057943-1.pdf]

Supplementary material: *Evaluation of HDL modulating interventions for cardiovascular risk reduction using a systems pharmacology approach*

Table 1: model species

Description	Notation	Initial concentration	Unit
Lipid-poor ApoA-I (pre-β_1)	A_{lp}	6.359	mg/dl
ApoA-I on α-HDL	A_α	135.018	mg/dl
Cholesteryl ester in α-HDL	CE_α	35.095	mg/dl
Particle concentration of α-HDL	N_α	0.00147	mmol/dl
Cholesteryl ester in LDL	CE_{LDL}	81.933	mg/dl
Cholesteryl ester in VLDL	CE_{VLDL}	5.987	mg/dl

Table 2: model parameters

Description	Notation	Value	Unit
Molecular weight of ApoA-I	m_A	28500	g/mol
Molecular weight of cholesterol	m_C	386	g/mol
Synthesis rate of ApoA-I	r_{in}^{lp}	28.459	mg/dl/day
Rate of kidney elimination	k_{kidney}	2.418	1/day
Dissociation rate of excess ApoA-I	k_{dissoc}	170.45	1/day
Rate constant in the lipidation of lipid-poor ApoA-I via ABCA1	k_{ABCA1}	95.179	1/day
Stoichiometry of FC to ApoA-I in nascent discs	γ	10.17	dimensionless
Rate constant of CE transfer: HDL to VLDL	k_{HV}^{CETP}	1.493	1/day
Rate constant of CE transfer: HDL to LDL	k_{HL}^{CETP}	6.924	1/day
Rate constant of CE transfer: LDL to HDL	k_{LH}^{CETP}	2.885	1/day
Rate constant of CE transfer: VLDL to LDL	k_{VL}	7.703	1/day
Synthesis rate of CE to VLDL	r_{in}^{VLDL}	1.504	mg/dl/day
Rate constant of CE elimination from VLDL	k_{out}^{VLDL}	1.298	1/day
Rate constant of CE elimination from LDL	k_{out}^{LDL}	0.644	1/day
Rate constant of SRB1-mediated CE elimination from HDL	k_{SRB1}^{HDL}	0.596	1/day
Constant contribution to the rate of α-HDL holo-particle uptake	k_{holo}^{c}	0.134	1/day
Size-dependent contribution to the rate of α-HDL holo-particle uptake	k_{holo}^{l}	-0.0159	1/nm/day
Parameter governing the particle concentration dependence of fusion rate	k_f	50000	1/(mmol/dl)

Table 3: summary of fluxes

Description	Expression	Rate	Unit
Flux 1: ApoA-I synthesis	r_{in}^{lp}	28.459	mg/dl/day
Flux 2: CE flux into plasma initiated by interaction of lipid-poor ApoA-I with ABCA1: the model RCT rate	$\gamma \times m_C/m_A \times k_{ABCA1}A_{lp}$	83.367	mg/dl/day
Flux 3: ApoA-I flux from lipid-poor ApoA-I to α-HDL	$k_{ABCA1}A_{lp}$	605.243	mg/dl/day
Flux 4: Regeneration of lipid-poor ApoA-I via HDL remodeling	$k_{dissoc}m_A\, N_\alpha(\frac{A_\alpha}{m_A \times N_\alpha} - n_{\text{ApoA-I}}^{\text{Shen}}(CE_\alpha/(m_C \times N_\alpha)))^\dagger$	592.16	mg/dl/day
Flux 5: Kidney removal of lipid-poor ApoA-I	$k_{kidney}A_{lp}$	15.377	mg/dl/day
Flux 6: Fusion of nascent spherical particles with mature α-HDL	$\frac{k_{ABCA1}A_{lp}}{2\, m_A}\frac{k_f N_\alpha}{1 + k_f N_\alpha}$	0.0105	mmol/dl/day
Rate 7: HDL particle holo-uptake rate constant	$k_{holo}(d) = k_{holo}^c + (d - 7.0nm) \times k_{holo}^l$	0.0969	1/day
Flux 8: SR-B1 mediated removal of CE from α-HDL	$k_{SRB1}^{HDL}CE_\alpha$	20.903	mg/dl/day
Flux 9: Synthesis of CE in VLDL	r_{in}^{VLDL}	1.504	mg/dl/day
Flux 10: Elimination of CE from VLDL	$k_{out}^{VLDL}CE_{VLDL}$	7.770	mg/dl/day
Flux 11: Elimination CE from LDL	$k_{out}^{LDL}CE_{LDL}$	52.773	mg/dl/day
Flux 12: Conversion and movement of VLDL-CE to LDL-CE via both lipolysis and CETP	$k_{VL}CE_{VLDL}$	46.122	mg/dl/day
Flux 13: CETP mediated CE transfer from HDL to VLDL	$k_{HV}^{CETP}CE_\alpha$	52.388	mg/dl/day
Flux 14: CETP mediated CE transfer from HDL to LDL	$k_{HL}^{CETP}CE_\alpha$	242.988	mg/dl/day
Flux 15: CETP mediated CE transfer from LDL to HDL	$k_{LH}^{CETP}CE_{LDL}$	236.337	mg/dl/day

†The functional dependence of the number of ApoA-I per particle according to the Shen's relation, $n_{\text{ApoA-I}}^{\text{Shen}}(x)$, is given in equations (10-13) of the reference Lu *et al* (2014).

Text 1: Equation system

$$\frac{dA_{lp}}{dt} = \text{(Flux 1)} - \text{(Flux 3)} - \text{(Flux 5)} + \text{(Flux 4)}$$

$$\frac{dA_\alpha}{dt} = \text{(Flux 3)} - \text{(Flux 4)} - (\text{Rate 7} \times A_\alpha)$$

$$\frac{dN_\alpha}{dt} = \frac{k_{ABCA1}A_{lp}}{2\, m_A} - \text{(Flux 6)} - (\text{Rate 7} \times N_\alpha)$$

$$\frac{dCE_\alpha}{dt} = \text{(Flux 2)} + \text{(Flux 15)} - \text{(Flux 13)} - \text{(Flux 14)} - \text{(Flux 8)} - (\text{Rate 7} \times CE_\alpha)$$

$$\frac{dCE_{LDL}}{dt} = \text{(Flux 12)} + \text{(Flux 14)} - \text{(Flux 15)} - \text{(Flux 11)}$$

$$\frac{dCE_{VLDL}}{dt} = \text{(Flux 9)} + \text{(Flux 13)} - \text{(Flux 12)} - \text{(Flux 10)}$$

Text 2: Intervention representation

1. Up-regulation of ApoA-I synthesis: increase r_{in}^{lp}
2. Infusion of rHDL: a bolus input to A_{lp}
3. Infusion of delipidated HDL: described below
4. Up-regulation of ABCA1: increase k_{ABCA1}

Table 4: LMK model calibration and validation data

Model Calibration	Model Validation
Clinical data (HDL-C, ApoA-I, LDL-C, VLDL-C) for hetero- and homozygotes of CETP deficiency<br>• Figure 2, Tables 6 & 7: Lu *et al* (2014)	Clinical data (HDL-C and ApoA-I) for hetero- and homozygotes of ABCA1 deficiency<br>• Figure 5: Lu *et al* (2014)
Dependence of ApoA-I clearance on HDL particle size<br>• Figure 4: Lu *et al* (2014)	Clinical data (HDL-C and ApoA-I) for hetero- and homozygotes of ApoA-I deficiency<br>• Figure 5: Lu *et al* (2014)
Relationship between HDL particle size and the number of ApoA-I molecules per particle (Shen's relation)<br>• Mazer *et al* (2013)	Biphasic behaviour in the ApoA-I tracer kinetics study<br>• Figure 6: Lu *et al* (2014)
CE fluxes between HDL and ApoB containing particles particles<br>• Figure 3 and Table 8: Lu *et al* (2014)	

Supplementary figures:

Figure 1: Simulated profiles of lipid parameters for RVX-208 @ 150 mg dose

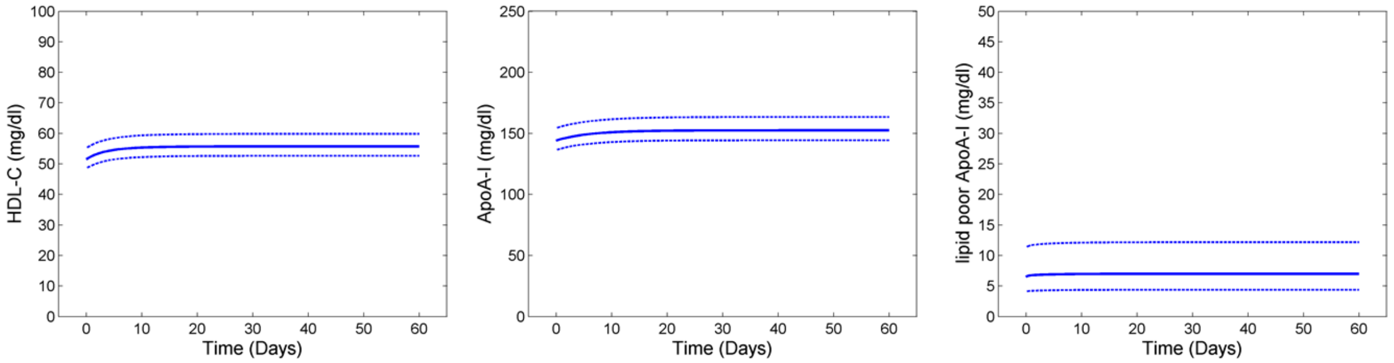

Figure 2: Simulated profiles of lipid parameters for CSL-111

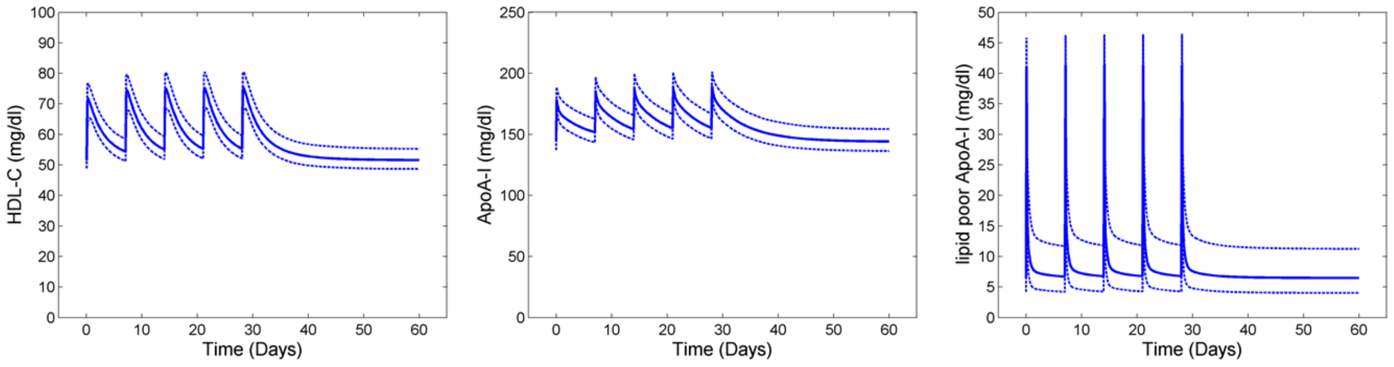

<u>Implementation of delipidation protocol</u>

The details of the delipidation protocol are described in (Waksman, Torguson et al. 2010); in this work the delipidation protocol is represented in 3 key steps:

(1) Plasma collection: in this step 1 liter of plasma is collected; this step takes 1.5 hours. In this step all lipid quantities in 1 liter of plasma are removed
(2) Delipidation: in this step the cholesterol from the HDL particles is removed with efficiency is ~80%. In this work this is represented as a removal of the cholesterol in 80% of the α-HDL particles. This removes all the cholesterol from the particles and apoA-I mass transfers from A_α to A_{lp}; the apoA-I (total) remained unchanged.
(3) Infusion: in this step the delipidated plasma is infused into the patient; this step takes 2 hours.

The changes in the total lipid quantities following each of the three steps in shown in the table below:

	initial steady state conc. (mg/dl)	initial steady state amount (mg)*	amount (mg) removed in plasma collection (step 1)*	amount (mg) remaining in patient after plasma collection (step 1)	amount (mg) after delipidation of collected plasma (step 2)	amount (mg) in patient after reinfusion (steps 3)	post delipidation conc. (mg/dl)
HDL-C	52.63	263.15	86.84	176.31	17.37	193.68	38.74
lipid poor ApoA-I	6.36	31.80	10.49	21.31	188.56	209.87	41.97
ApoA-I on aHDL	134.90	674.50	222.59	451.92	44.52	496.43	99.29
ApoA-I (total)	141.26	706.30	233.08	473.22	233.08	706.30	141.26

*typical volume of 5 liters of blood and 3 liter of plasma used

The dynamics of the patient's lipid profiles during this process are not included in the model; instead the changes in the concentrations before and after the complete process are implemented. The patient's responses to the changes in the lipid parameters with each delipidation process for longer more relevant time scales in shown in Figure 4 of the manuscript.

References

Lu, J., K. Hubner, et al. (2014). "An in-silico model of lipoprotein metabolism and kinetics for the evaluation of targets and biomarkers in the reverse cholesterol transport pathway." <u>PLoS Comput Biol</u> **10**(3): e1003509.

Mazer NA, Giulianini F, et al. (2013). "A comparison of the theoretical relationship between HDL size and the ratio of HDL cholesterol to apolipoprotein A-I with experimental results from the Women's Health Study." <u>Clin Chem</u> 59: 949–958.

Waksman, R., R. Torguson, K. M. Kent, A. D. Pichard, W. O. Suddath, L. F. Satler, B. D. Martin, T. J. Perlman, J. A. Maltais, N. J. Weissman, P. J. Fitzgerald and H. B. Brewer, Jr. (2010). "A first-in-man, randomized, placebo-controlled study to evaluate the safety and feasibility of autologous delipidated high-density lipoprotein plasma infusions in patients with acute coronary syndrome." J Am Coll Cardiol **55**(24): 2727-2735.
